# Native range climate influences nonstructural carbohydrate storage in oak species growing in a common garden

**DOI:** 10.1007/s00442-025-05773-6

**Published:** 2025-07-11

**Authors:** Kyra A. Prats, Josephine Brigham, Levi Berry, Dylan K. Wainwright, Morgan E. Furze

**Affiliations:** 1 Department of Botany and Plant Pathology, Purdue University, West Lafayette, IN 47907 USA; 2 Center for Plant Biology, Purdue University, West Lafayette, IN 47907 USA; 3 Department of Biological Sciences, Purdue University, West Lafayette, IN 47907 USA; 4 Department of Forestry and Natural Resources, Purdue University, West Lafayette, IN 47907 USA

**Keywords:** Climate, Local adaptation, Nonstructural carbohydrates, Oaks, Phylogenetic comparative methods, *Quercus*

## Abstract

Global climate change and related environmental stress threaten the survival of long-lived tree species. To ensure survival in increasingly unfavorable environments, trees need to either acclimate through phenotypic plasticity, adapt via genetic differentiation, or migrate to more favorable climates altogether. Nonstructural carbohydrate (NSC) storage is a critical trait that supports proper metabolic function in plants and has been shown to prolong their survival in the face of stress. However, relatively few studies have explored local adaptation of NSC storage, with none to our knowledge focusing on oaks, an abundant and ecologically important genus primarily across the Northern Hemisphere. Here, we used a common garden of oak species to assess whether NSC storage is locally adapted by exploring whether species’ native range climate influences NSC storage in the stem. We found that the proportion of soluble sugars, the proportion of starch, and starch concentration were influenced by native range temperature, such that species from hotter regions had a larger proportion of soluble sugars, smaller proportion of starch, and lower starch concentration than species from colder regions. These correlations suggest that some NSC traits (i.e., proportion of sugars/starch) are genetically controlled in the genus *Quercus*. Our study has implications for how different oak species may respond to and survive under environmental stress and changing climates.

**Supplementary Information:**

The online version contains supplementary material available at 10.1007/s00442-025-05773-6.

## Introduction

Environmental stress poses a major threat for trees under current and future climatic conditions, driving forest ecosystem decline and jeopardizing biodiversity and ecosystem services (Allen et al. 2010; Anderegg et al. 2016; McDowell et al. 2018). As global change proceeds, long-lived trees will need to migrate to more favorable conditions, acclimate through phenotypic plasticity, or adapt via genetic differentiation, or use a combination of these strategies to persist (Mimura and Aitken 2007; Franks 2011; Sork et al. 2013). Local adaptation of functional traits has occurred in many forest trees due to selective factors like climate (Linhart and Grant 1996; Kawecki and Ebert 2004; Alberto et al. 2013), and strong support for variation in traits along environmental gradients has been found (Alberto et al. 2013). For instance, the phenology and growth traits of temperate and boreal tree species have locally adapted genetic variation following temperature, elevational, or latitudinal clines/gradients (Campbell 1979; Rehfeldt 1995; St Clair et al. 2005; Mimura and Aitken 2007; Savolainen et al. 2007; Prats et al. 2019). Tree survival will depend on such adaptive traits that allow trees to withstand changing environments.

Nonstructural carbohydrate (NSC) storage is one such trait that may provide a survival advantage to trees facing abiotic and biotic stress. NSCs consist of soluble sugars and insoluble starch which serve as substrates for growth, respiration, defense, and osmoregulation (Chapin 1990; Dietze et al. 2014; Hartmann and Trumbore 2016). Further, NSCs can be allocated to storage and then remobilized to support critical tree processes during times when photosynthesis is limited, thus supporting predictable periods when demand exceeds supply, as well as providing resilience against more unpredictable periods of stress (Landhaüsser and Lieffers 2012; Sala et al. 2012; Brien et al. 2014; Sevanto et al. 2014; Adams et al. 2017; Barker Plotkin et al. 2021). While starch is a long-term storage molecule, soluble sugars, such as glucose, sucrose, fructose, and raffinose, serve as critical osmolytes (Yuanyuan et al. 2009; Blumstein et al. 2023; Long and Adams 2023). Drought stress activates enzymes that degrade starch into soluble sugars which then lower osmotic potential and maintain cell turgor (Thalmann and Santelia 2017; Blum 2017; Guo et al. 2020; Aranda et al. 2021). Similarly, soluble sugars can improve resistance to heat stress (Hüve et al. 2006; Gangola and Ramadoss 2018; Parker et al. 2020) and, on the opposite end of the temperature spectrum, soluble sugars prevent tissue damage during freezing stress (Thomashow 1999; Thalmann and Santelia 2017). Therefore, given the central role of sugars in the response to stress like drought, trees from environments that are on average hotter-drier than others might be expected to invest more to NSC storage (Wiley and Helliker 2012). Such an increase in total NSC storage could be a result of evolution (Blumstein et al. 2020) or a plastic response (Piper et al. 2017). Trees in hotter-drier environments may also invest a larger fraction of NSC stores as soluble sugars for osmotic regulation (Blumstein et al. 2023).

However, despite the importance of NSCs in tree resilience, relatively few studies have examined local adaptation of NSC storage, and none to our knowledge have focused on oaks. Long-lived and sessile organisms like oak trees may be able to physiologically adjust their traits over both short (plasticity) and long (local adaptation) timescales. Determining the extent of plastic and genetic variation in such adaptive traits is crucial for predicting whether populations will be able to evolve to keep pace with environmental change (Duputié et al. 2015; Hendry 2016). For example, plastic variation may help trees acclimate to environmental stress in the short term but may ultimately not be enough to support long-term survival. In addition, adaptive evolution may be limited by genetic variation, leading to local extinction (Alberto et al. 2013; Borkowski et al. 2017).

Common gardens provide a powerful tool to study the local adaptation of functional traits and can be used to assess the extent to which NSCs are locally adapted in trees (Alberto et al. 2013). Since environmental variation is minimized, a common garden setting allows the influence of genetics to be ascertained. Variation in NSC storage within a common garden environment can provide insight into whether trees are locally adapted to variation in environmental stress across their native ranges and if adaptive evolution exists as a potential strategy to deal with increased stress. Results from previous common garden experiments assessing NSC-climate relationships have been somewhat equivocal. One common garden study demonstrated that *Populus trichocarpa* trees originating from hotter-drier environments stored more NSCs (Blumstein et al. 2020), whereas another study demonstrated that *Tamarix chinensis x Tamarix ramosissima* trees from higher elevations with colder conditions stored more NSCs (Long et al. 2021). Often, the focus has been on a single species; thus, there exists an opportunity to compare NSC storage within a genus at a broader evolutionary scale and, more specifically, to assess the influence of species’ native range climate on NSC storage. The genus *Quercus* (oaks) dominates North America, Mesoamerica, and Eurasia (Nixon 1997; Hipp et al. 2018; Cavender-Bares 2019), with the largest biomass and species diversity of any forest tree genus in the United States and Mexico (Cavender-Bares 2016). Oaks inhabit a range of ecologically diverse environments across elevations and climates and are consequently major drivers of forest ecosystem function (Hipp et al. 2018); understanding their response to global change is therefore a priority.

Using a common garden of oak species, we assessed whether there was a signature of local adaptation in NSC storage traits by exploring whether species’ native range climate (including metrics of precipitation, temperature, and aridity) influences NSC storage in the stem, a major storage organ (Furze et al. 2018). We hypothesized that NSC storage may reflect local adaptation to species’ native range climates such that variation in NSC storage among species in the common garden would be correlated with native range climate. Specifically, we expected that if total NSC storage and/or the proportion of soluble sugars increases tree survival in the face of stressful periods, then both traits would be larger in oak species originating from hotter and drier native ranges.

## Materials and methods

### Common garden sampling of oak species

Samples were collected from 22 oak species at the Peter J. Shields Oak Grove of the University of California Davis Arboretum and Public Garden (Davis, CA, USA) in September 2019, with species having deciduous (n = 10), brevideciduous (n = 3), and evergreen (n = 9) leaf habits. 

Comparing several *Quercus* species growing in a common garden setting minimizes environmental factors and allows for exploration of local adaptation on NSC storage. Oak trees were planted in the collection from 1963 to 1980, and collection and sampling information for each individual tree are provided (Table S1). We sampled 1–3 individual trees per species; there was only a single tree for *Q. tomentella* and *Q. crassifolia*. A stem core was collected from each tree by carefully chiseling away the outermost stem tissues to expose the xylem and then a 2-mm diameter microcore (Trephor, 15 mm length, University of Padua, Italy; Rossi et al. 2006) was used to obtain a core. Both tools were sterilized with 70% ethanol between samples. Stem cores were kept on dry ice in the field and then stored at − 80 °C. Samples were dried prior to grinding and NSC analysis.

### NSC measurements from oak species in the common garden

We measured sugar and starch concentrations (mg per g dry wood) for stem cores using a standardized protocol (Landhäusser et al. 2018). Stem cores were dried in a drying oven at 100 °C for 1 h to deactivate starch degrading enzymes, and then at 70 °C for 2–3 days until the samples were fully dry. Samples were then ground (mesh 20; Thomas Scientific Wiley Mill, Swedesboro, NJ, USA; SPEX SamplePrep 5100 Mixer Mill, Metuchen, NJ, USA) and at least 10 mg of each sample was weighed out for sugar and starch analyses.

To obtain sugar concentrations, ground samples were extracted with hot ethanol and soluble sugar extracts underwent a reaction with phenol–sulfuric acid, with the resulting color solution measured at 490 nm on a spectrophotometer (Spectronic 20 Genesys, 4001/4; Spectronic Instruments, Rochester, NY, USA). The tissue was then digested with α-amylase and amyloglucosidase to obtain starch concentrations. A peroxidase-glucose oxidase color reagent was added, and the resulting color solution was measured at 525 nm on the spectrophotometer. Both sugars and starch were expressed on the same scale (i.e., glucose equivalents) and were summed together to obtain total NSC concentration. We then calculated the proportion of total NSCs that were sugars versus starch as sugar concentration/total NSC concentration (i.e., proportion of soluble sugars) and starch concentration/total NSC concentration (i.e., proportion of starch), respectively. Thus, the NSC traits for each tree were stem sugar concentration (mg g^−1^), stem starch concentration (mg g^−1^), stem total NSC concentration (mg g^−1^), proportion of soluble sugars (%), and proportion of starch (%). NSC data for individual trees are provided in Table S2. An internal laboratory standard was included in each set of samples processed for NSCs with a sugar concentration of 35.6 ± 0.9 (mean ± SD) and starch concentration of 22.8 ± 1.5 (mean ± SD.), respectively.

### Native range

For half of the study species, their native range was obtained as a shapefile from the United States Tree Atlas repository (Little 1971, 1976; Petry and Taylor 2022). For the other half of the study species, no such shapefiles existed to our knowledge. Thus, for all 22 oak species, we first obtained the native range locations for each species using the Kew Royal Botanic Gardens “Plants of the World Online” database (POWO 2025). The states, regions, and countries of each species’ native range were noted (Table S3). Then, we used the get_gbif() function from the gbif.range R package to access the Global Biodiversity Information Facility (GBIF) database to obtain occurrence data (i.e., individual tree records) within the native range of each species based on the locations noted from”Plants of the World Online” database (GBIF.org 2025). Occurrences were selected and filtered by basis of record (‘Preserved specimen’), location (‘Including coordinates’), and year (2018–2023); occurrence data were limited to the last five years to ensure accurate georeferencing. The get.range() function was then used to generate a shapefile for the native range of each species. A shapefile for *Q. garryana* could not be generated in this way because it did not meet the minimum occurrence requirements to estimate the native range. Thus, 11 species had two native range shapefiles (one obtained from the United States Tree Atlas and one generated from GBIF), 10 species had a single shapefile generated from GBIF, and one species (*Q. garryana*) had a single shapefile obtained from the United States Tree Atlas (Table S3).

### Climate data

To examine the native range climate of the oak species, we averaged climate data across the entire native range of each species. Temperature and precipitation data were obtained from the gridded Climate Research Unit Timeseries (CRU TS) data v.4.06, which is gridded to a 0.5 × 0.5-degree resolution of monthly climate data from 1901 to 2021 (Harris et al. 2020). Data obtained for each month were total precipitation (pre; mm), minimum temperature (tmn; °C), maximum temperature (tmx; °C), and average temperature (tmp; °C). We then used these CRU data to calculate average annual conditions for each tree occurrence from 1901 through September 2019 (the month and year when we sampled trees in the common garden). Average annual precipitation (mm) for each tree was calculated by first summing total precipitation for each year and then averaging across years. Average annual minimum, average annual maximum, and average annual temperatures (°C) were calculated by taking the mean of the minimum, maximum, and average temperatures, respectively, over the entire period. Additionally, to assess aridity, we obtained Global Aridity Index (AI) data, with 30 arc second resolution, which provided an annual average over the entire period 1970–2000. For each of the 11 species with a single shapefile of its native range, climate metrics were averaged across the entire native range and used in phylogenetic principal components analysis (PPCA) (Table S4). For each of the remaining 11 species that had two shapefiles of its native range (one from United States Tree Atlas and one from GBIF), climate metrics were averaged across the entire native range in each shapefile and then were averaged together to get a species mean for each climate metric to be used in PPCA (Tables S4). Correlations between climate variables were assessed prior to PPCA (Table S5).

### PPCA of annual climate data and regression

We used PPCA to summarize the major axes of variation in annual climate data using the phyl.pca() function from the phytools package (version 1.2–0) in R (version 4.2.2) (Revell 2012; R Core Team 2021). The phylogenetic model used for the error term was Pagel's λ (lambda). NSC data from individual trees in Table S2 were used to calculate species’ means. To examine the relationship between native range climate and NSC traits of species growing in the common garden, the first principal component (PC1) and the second principal component (PC2) scores were extracted and independently used for phylogenetic linear regression with the species’ means of each NSC trait. Phylogenetic linear regression was performed using the phylolm() function from the phylolm package (version 2.6.2) and evaluated with a significance level of α = 0.05. Further, to assess whether oak species differed in PPCA space based on leaf habit, we performed a permutational multivariate analysis of variance (PERMANOVA) based on Euclidean distance with 999 permutations using the adonis2() function from the vegan R package.

## Results

### PPCA of native range climate

**Fig. 1:**
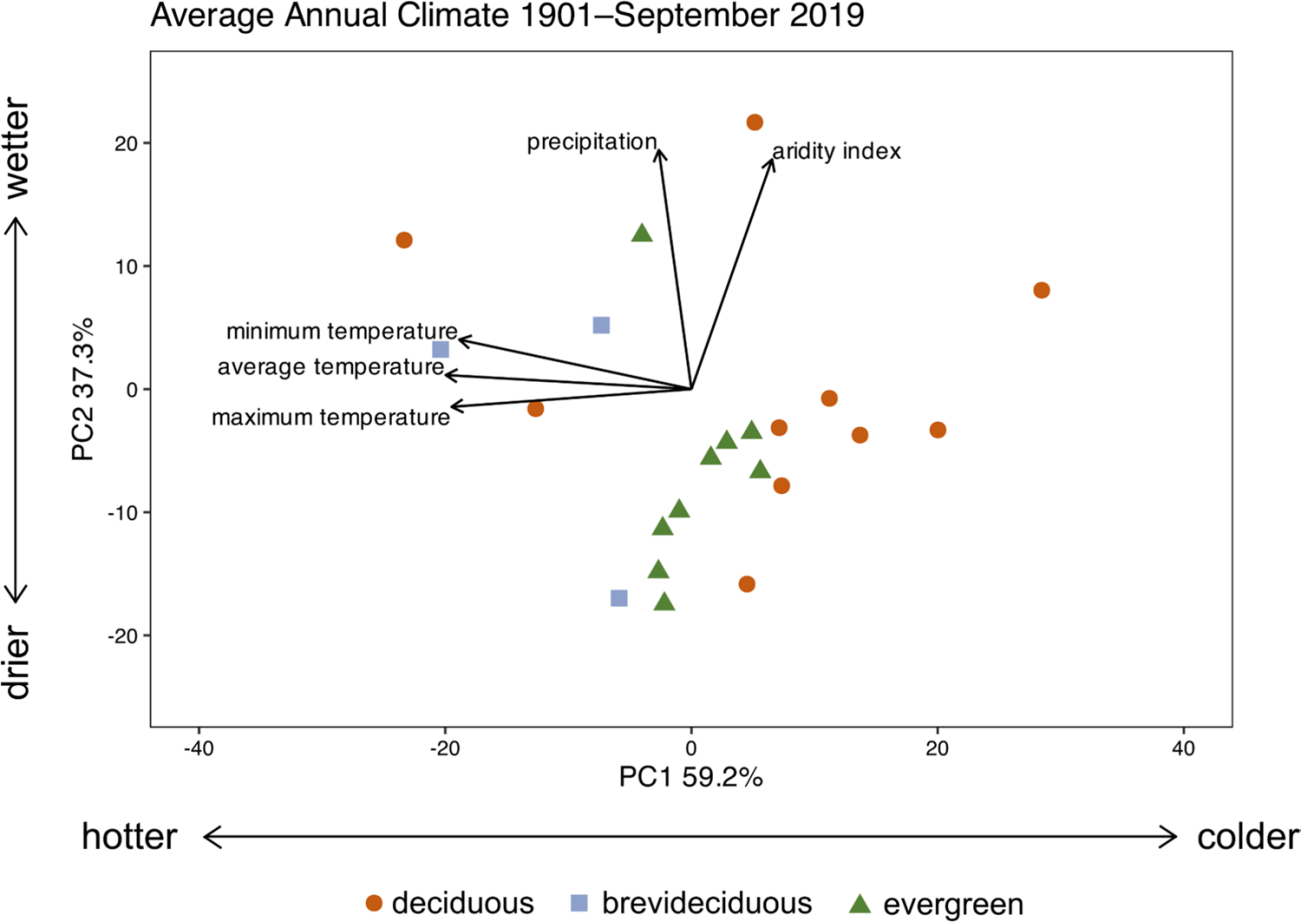
Phylogenetic principal component analysis (PPCA) of average annual climate variables of species’ native ranges between 1901 and September 2019. PC1 (a gradient from hotter (negative scores) to colder (positive scores) temperatures) and PC2 (a gradient from drier (negative scores) to wetter conditions (positive scores) explain 96.5% of the variance. Points are species’ means (n = 22 species) with color and shape indicating leaf habit as follows: deciduous (orange circle), brevideciduous (blue square), and evergreen (green triangle)

Together, PC1 and PC2 explained 96.5% of the variation in annual climate data (Fig. 1). PC1 explained 59.2% of the variation in annual climate data and described a gradient from hotter (negative scores) to colder (positive scores) temperatures. PC2 explained 37.3% of the variation in annual climate data and described a gradient from drier (negative scores) to wetter conditions (positive scores). Leaf habit did not explain a significant portion of the variation in PC1 and PC2 scores (F = 2.21, R^2^ = 0.19, P = 0.09).

### Relationship between PC1 and PC2 and traits measured on oak species in the common garden

**Fig. 2:**
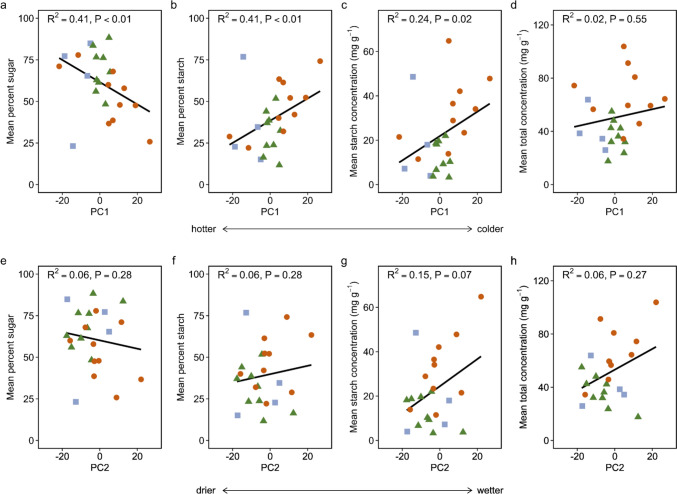
Relationship between PC1 and **a** the proportion of NSC stores as soluble sugars in the stem, **b** the proportion of NSC stores as starch in the stem, **c** starch concentration in the stem, and **d** total NSC concentration in the stem, as well as the relationship between PC2 and **e** the proportion of NSC stores as soluble sugars in the stem, **f** the proportion of NSC stores as starch in the stem, **g** starch concentration in the stem, and **h** total NSC concentration in the stem. PC1 describes a gradient from hotter (negative scores) to colder (positive scores) temperatures in **a**–**d**. PC2 describes a gradient from drier (negative scores) to wetter conditions (positive scores) in **e**–**h**. Individual points are species’ means (n = 22 species), with color and shape indicating leaf habit as follows: deciduous (orange circle), brevideciduous (blue square), and evergreen (green triangle)

PC1 was negatively correlated with the proportion of soluble sugars in the stem (Fig. 2a), and positively correlated with the proportion of starch (Fig. 2b) and starch concentration in the stem (Fig. 2c). Native range temperature loaded strongly on PC1, indicating that as temperature decreased, the proportion of soluble sugars decreased and the proportion of starch and starch concentration increased. All other NSC traits measured for the species in the common garden (i.e., total and sugar concentrations) were not significantly correlated with PC1 (total concentration Fig. 2d; sugar concentration Fig. S1a). A dry to wet gradient loaded strongly on PC2, but PC2 was not significantly correlated with any NSC traits measured on the oak species in the common garden (Fig. 2e–h; Fig. S1b).

## Discussion

### Native range temperature is related to NSC storage in tree stems

We found evidence partially supporting our hypothesis that stem NSC storage correlates with native range climate when species are grown in a common garden. However, while we hypothesized that both total NSC storage and the proportion of soluble sugars in the stem would be higher in oak species originating from hotter and drier native ranges, we found that the proportion of soluble sugars was only significantly influenced by temperature (PC1). Additionally, we found that the proportion of starch and starch concentration were also significantly influenced by temperature (PC1). We observed that species from hotter regions had a larger proportion of soluble sugars, smaller proportion of starch, and lower starch concentration than species from colder regions.

Native range aridity (PC2) was not correlated with any measured NSC traits. This was somewhat surprising given that hot and dry are often intrinsically linked environmental conditions. However, our finding is supported by recent work which revealed that NSCs were associated with global clinal patterns in temperature, but not aridity; that is, there was no evidence for evolved differences between species in terms of their maximum NSC storage in relation to aridity (Blumstein et al. 2023). Thus, both studies suggest that temperature may be the primary factor that shapes NSC dynamics. The lack of a relationship between NSCs and aridity in our study could also be due to the fact that oak species have different strategies for facing arid conditions that may not rely on NSC storage, like leaf shedding or deep roots, leading to high variation in our data. Further, aridity is a more complex metric than temperature. It takes into account precipitation and potential evapotranspiration, so this may make it more difficult to detect consistent aridity-NSC relationships across diverse species.

Moreover, in the aforementioned global, multi-species meta-analysis, Blumstein et al. (2023) looked at species-level patterns of NSC traits versus the average climate at the collection location, and did not find a relationship between the proportion of sugars in the stem and climate, but they did find that sugar and total NSC concentrations were higher in trees originating from hotter locations. In contrast, within a single genus in a common garden, our results suggest that the proportion of sugars in the stem was higher in species originating from hotter native ranges. This discrepancy not only showcases the importance of considering within versus between genus patterns, but also highlights the fact that relationships do not often hold at every scale of examination (Anderegg et al. 2018). For example, although our results suggest that certain aspects of NSC storage variation are adaptive, there is still plasticity in traits (Cavender-Bares 2019) like NSCs or those that may influence NSCs, and plasticity within a genus may mask evolved climate-NSC trait correlations at broader taxonomic scales. Conversely a multi-genus comparison might average across disparate life histories and storage strategies, potentially obscuring patterns that might be clearer when assessing a single genus like *Quercus*.

### Proportion of stem sugar is higher in species originating from hotter native ranges, while the proportion of stem starch is higher in species originating from colder native ranges

Soluble sugars play a key role in maintaining function, like turgor, hydraulic conductivity, and tissue integrity, under conditions that may be very hot, cold, or dry (Kaplan and Guy 2004; Sevanto et al. 2014; Guo et al. 2020; Sapes et al. 2021; Tomasella et al. 2021). In environments that are on average hotter and drier, selection may favor allocation to NSC storage, which can then support critical survival functions such as by serving as a carbon starvation buffer or osmotic buffer. Few studies have examined whether plants evolve higher soluble sugar content for osmoregulation (Blumstein and Hopkins 2020; Long et al. 2021; Reyes-Bahamonde et al. 2021; Piper and Fajardo 2023). Our results suggest that oak species from hotter climates invest in a higher proportion of their NSC stores as soluble sugars compared to those from colder climates. This larger investment into an osmotic buffer may provide an advantage that allows them to better survive and persist through stressful periods. Notably, total NSC concentration was not related to native range temperature, suggesting that the overall amount of reserves may be less important than investment in a larger sugar buffer. Reserves may not be equally accessible to support metabolism (Hartmann and Trumbore 2016; Hartmann et al. 2020)—they are distributed across shallow and deep tissues and comprise a mix of new and old C—and older, deeper reserves may be unavailable or only accessible following chronic stress (Peltier et al. 2023). Thus, larger reserves may not equate to increased resilience to stress, while the proportion of sugar available to serve as an osmotic buffer may be what is ultimately important for proper function under hotter conditions.

On the other end of the temperature spectrum, cold conditions require plants to use NSC stores as osmolytes for freezing tolerance (Thalmann and Santelia 2017). While oak species originating from colder native ranges did not have higher total concentrations or a larger proportion of sugars in the stem, they did have a larger proportion of starch and higher starch concentration in the stem. Since starch is considered a longer-term storage molecule, oak species from colder native ranges may have evolved to store more starch on average to buffer against the long, harsh dormancy period or to restart growth quickly in the spring. Starch stores can be converted into sugars during dormancy, whereby sugars are then used to support wintertime respiration and can serve as osmolytes to prevent freezing.

The correlations between NSC traits and species’ native range temperature in the genus *Quercus* suggest that NSC storage, specifically the proportion of stores as sugars and starch, is shaped by species-level differences that are maintained across environments. Variation in phenotypic traits is thought to arise from heritable factors (González-Martínez et al. 2006; De Villemereuil et al. 2016), and NSC storage has been shown to be heritable and locally adapted in *Populus trichocarpa* trees (Blumstein et al. 2020). Common garden studies have highlighted the effects of species’ native range climate on a variety of physiological, anatomical, and morphological characteristics of plants, including xylem (Fichot et al. 2009; Schreiber et al. 2015; Bourne et al. 2017) and hydraulic traits (Vander Willigen et al. 2000; Bourne et al. 2017), leaf water relations (Dudley 1996; Baltzer et al. 2008), leaf morphology traits (Ramírez-Valiente et al. 2010; Garot et al. 2021), stomata behavior traits (Bourne et al. 2017), and, notably, NSC reserves (Blumstein et al. 2020; Long et al. 2021; Piper and Fajardo 2023). These results suggest that local adaptation can involve numerous wide-ranging traits, from the physiological to the anatomical, and our findings are consistent with local adaptation, highlighting some support for genetic control of NSC traits in the genus *Quercus*. If sugar and starch proportions are genetically controlled as suggested by our results, environmental stress may require trees to genetically adapt their NSC physiology to changes in climate. Genetic adaptation can be a slow process for long-lived species like oaks, especially in the face of the rapid pace of climate change, so changing NSC allocation strategies in the face of relatively rapid changes in the environment may also rely on plasticity in these traits.

While our study advances understanding of local adaptation in *Quercus* trees, it has some limitations. First, we sampled at a single point in time from a single organ which does not fully capture seasonal or spatial variation in NSC storage (Martínez-Vilalta et al. 2016). However, tree stems are a major storage organ, comprising ~ 40% of NSC reserves (Richardson et al. 2015), and we collected stem tissue when NSC reserves were expected to be at their annual maximum (Furze et al. 2019). Second, since we assessed NSCs across the *Quercus* genus rather than across genotypes of a single species, we cannot account for confounding factors and inherent differences between *Quercus* species in the common garden, such as tree age and maternal effects, that may influence NSC reserves (Kawecki and Ebert 2004; Furze et al. 2021; Wang et al. 2023). Nevertheless, a previous study also found support for local adaptation in plant traits across species within the *Eucalyptus* genus (Bourne et al. 2017) and comparing species from the same genus growing in a common garden allows both environmental and phylogenetic variation to be minimized.

## Conclusion

We assessed variation in NSC storage across oak species growing in a common garden. By measuring species from a single genus in the same location, we controlled for environmental and phylogenetic variation and found that the proportion of stores as sugars and the proportion of stores as starch—inverse measurements capturing total NSCs in the stem—were correlated with native range temperature. In this way, species’ native range temperature shaped their responses to the common garden environment to yield maintained differences in NSC reserves, which provides evidence that NSC reserves may be genetically constrained. This work informs how different oak species may respond to environmental stress and has implications for tree survival under changing climates.

## Supplementary Information

Below is the link to the electronic supplementary material.

Supplementary file1 (DOCX 170 KB)

## Data Availability

The data that supports the findings of this study are available in the Supplementary Information of this article.
