# Supplementary material for: Native range climate influences nonstructural carbohydrate storage in oak species growing in a common garden
Source: Oecologia. 2025 Jul 11;207(8):132. doi: 10.1007/s00442-025-05773-6 (PMC12254077; doi:10.1007/s00442-025-05773-6)

**Supplementary Materials**

**Title:** Native range climate influences nonstructural carbohydrate storage in oak species growing in a common garden

**Journal:** *Oecologia*

**Authors:** Kyra A. Prats, Josephine Brigham, Levi Berry, Dylan K. Wainwright, Morgan E. Furze

**Corresponding author:** Purdue University, mfurze@purdue.edu

**Table S1.** Collection information for individual trees of 22 oak species sampled in September 2019 at the Peter J. Shields Oak Grove of the University of California Davis Arboretum and Public Garden (Davis, CA, USA). Tree tag is the tag number on each tree. Scientific name is the genus and species of each tree. Accession number and collection information were provided by the arboretum. The age of trees in the study is 53 ± 4 years (mean ± SD).

| tree tag | accession number | scientific name | collection information (e.g., year, origin, locality) |
| --- | --- | --- | --- |
| 14 | A64.0713 | *Quercus agrifolia* Née | 1964; University of California Berkeley Botanical Garden; seedlings from trees native to Strawberry Canyon, Berkeley, CA, USA |
| 15 | A64.0713 | *Quercus agrifolia* Née | 1964; University of California Berkeley Botanical Garden; seedlings from trees native to Strawberry Canyon, Berkeley, CA, USA |
| 22 | A64.0713 | *Quercus agrifolia* Née | 1964; University of California Berkeley Botanical Garden; seedlings from trees native to Strawberry Canyon, Berkeley, CA, USA |
| 81 | A64.1304 | *Quercus cerris* L. | 1964; Royal Botanic Gardens, Kew, Richmond, United Kingdom |
| 82 | A64.1304 | *Quercus cerris* L. | 1964; Royal Botanic Gardens, Kew, Richmond, United Kingdom |
| 250 | A65.0013 | *Quercus chrysolepis* Liebm. | 1965; Banner Grade Canyon, 3000 ft, San Diego County, CA, USA |
| 251 | A65.0013 | *Quercus chrysolepis* Liebm. | 1965; Banner Grade Canyon, 3000 ft, San Diego County, CA, USA |
| 30 | A64.1324 | *Quercus coccifer*a L. | 1964; Barcelona, Spain |
| 31 | A64.1324 | *Quercus coccifer*a L. | 1964; Barcelona, Spain |
| 261 | A68.0355 | *Quercus crassifolia* Humb. & Bonpl. | 1966; 80 km marker on RN-l between Petzcia and Lago Atitlan, Guatemala |
| 205 | A64.1274 | *Quercus douglasii* Hook. & Arn. | 1964; 2.5 mi. below summit of Mix Canyon, Solano County, CA, USA |
| 215 | A64.0406 | *Quercus douglasii* Hook. & Arn. | 1964; Yuba River, CA, USA |
| 228 | A65.0011 | *Quercus engelmannii* E. Greene | 1965; Dry steam side, 500 ft, San Diego County, CA, USA |
| 230 | A65.0011 | *Quercus engelmannii* E. Greene | 1965; Dry steam side, 500 ft, San Diego County, CA, USA |
| 99 | A71.0132 | *Quercus garryana* Douglas ex Hook. var *garryana* | 1971; University of Utah Arboretum, Salt Lake City, Utah, USA |
| 100 | A71.0132 | *Quercus garryana* Douglas ex Hook. var *garryana* | 1971; University of Utah Arboretum, Salt Lake City, Utah, USA |
| 102 | A71.0132 | *Quercus garryana* Douglas ex Hook. var *garryana* | 1971; University of Utah Arboretum, Salt Lake City, Utah, USA |
| 233 | A63.0002 | *Quercus grisea* Liebm. | 1963; 27.3 mi SW of Silver City, 6000 ft, New Mexico, USA |
| 234 | A63.0002 | *Quercus grisea* Liebm. | 1963; 27.3 mi SW of Silver City, 6000 ft, New Mexico, USA |
| 33 | A64.1326 | *Quercus ilex* L. subsp. *ilex* | 1964; Barcelona, Spain |
| 53 | A64.1326 | *Quercus ilex* L. subsp. *ilex* | 1964; Barcelona, Spain |
| 44 | A64.1315 | *Quercus ilex* L. subsp. *ilex* | 1964; Royal Botanic Gardens, Kew, Richmond, United Kingdom |
| 292 | A64.1184 | *Quercus macrocarpa* Michx. | 1964; Dominion Arboretum & Botanic Garden, Ottawa, Ontario, Canada |
| 293 | A64.1184 | *Quercus macrocarpa* Michx. | 1964; Dominion Arboretum & Botanic Garden, Ottawa, Ontario, Canada |
| 256 | A68.0354 | *Quercus oleoides* Schl. & Cham. | 1966; West edge of town, Masaguara, Honduras |
| 257 | A68.0354 | *Quercus oleoides* Schl. & Cham. | 1966; West edge of town, Masaguara, Honduras |
| 54 | M05.8009 | *Quercus phillyreoides* A. Gray | 1976; Royal Botanic Gardens, Kew, Richmond, United Kingdom |
| 56 | M05.8007 | *Quercus phillyreoides* A. Gray | 1976; Royal Botanic Gardens, Kew, Richmond, United Kingdom |
| 57 | M05.8008 | *Quercus phillyreoides* A. Gray | 1976; Royal Botanic Gardens, Kew, Richmond, United Kingdom |
| 207 | A63.0007 | *Quercus pungens* Liebm. | 1963; Pine Spring, 5700 ft, Culberson County, TX, USA |
| 235 | A63.0007 | *Quercus pungens* Liebm. | 1963; Pine Spring, 5700 ft, Culberson County, TX, USA |
| 306 | A64.1311 | *Quercus robur* L. subsp. *robur* | 1964; Royal Botanic Gardens, Kew, Richmond, United Kingdom |
| 307 | A81.0414 | *Quercus robur* L. subsp. *robur* | 1980; State Forest near Kandern, Germany |
| 26 | A65.0838 | *Quercus rugosa* Née | 1963; Huntington Botanical Gardens, San Marino, CA, USA; 3 km before El Oro on road from Atlacomulco, Mexico |
| 258 | A68.0360 | *Quercus rugosa* Née | 1966; 20 miles from Oaxaca on the road to Boone Halberg's finca near Ixtlan, Mexico |
| 68 | A64.0062 | *Quercus sinuata* Walt. var *sinuata* | 1963; Parking area in vicinity of concession building, Palmetto State Park, Gonzales County, TX USA |
| 69 | A64.0062 | *Quercus sinuata* Walt. var *sinuata* | 1963; Parking area in vicinity of concession building, Palmetto State Park, Gonzales County, TX USA |
| 65 | A64.1325 | *Quercus suber* L. | 1964; Barcelona, Spain |
| 79 | A64.1325 | *Quercus suber* L. | 1964; Barcelona, Spain |
| 80 | A64.1280 | *Quercus suber* L. | 1964; Quad area University of California Davis, Davis, CA, USA |
| 32 | A68.0609 | *Quercus tomentella* Engelm. | 1968; Bottom of gorge of tributary leading to the southernmost drainage to China Harbor, Santa Cruz Island, CA, USA |
| 248 | A64.1309 | *Quercus trojana* Webb. | 1964; Royal Botanic Gardens, Kew, Richmond, United Kingdom |
| 298 | A64.0008 | *Quercus trojana* Webb. | 1963; unknown |
| 264 | A69.0181 | *Quercus variabilis* Blume. | 1969; Aritaki Arboretum, Koshigaya-shi, Saitama- ken, Japan |
| 265 | A69.0181 | *Quercus variabilis* Blume. | 1969; Aritaki Arboretum, Koshigaya-shi, Saitama- ken, Japan |
| 58 | A64.0012 | *Quercus virginiana* Miller | 1963; Junction of Texas 1095 and 521, 13.8 mi. west of Wadsworth, TX, USA |
| 60 | A64.0012 | *Quercus virginiana* Miller | 1963; Junction of Texas 1095 and 521, 13.8 mi. west of Wadsworth, TX, USA |

**Table S2**. Stem NSC and LMA data from individual trees of the 22 oak species that were sampled in September 2019 at the Peter J. Shields Oak Grove of the University of California Davis Arboretum and Public Garden (Davis, CA, USA). There was only a single tree of *Q. tomentella* and *Q. crassifolia*, but we sampled them twice. We could only collect a single stem core from each for NSC measurements due to coring restrictions. We calculated the proportion of total NSCs that were sugars versus starch as sugar concentration/total NSC concentration (i.e., percent sugar below) and starch concentration/total NSC concentration (i.e., percent starch below), respectively.

| tree tag | accession number | species | stem sugar (mg g^-1^) | stem  starch  (mg g^-1^) | stem total NSC  (mg g^-1^) | percent sugar (%) | percent starch (%) |
| --- | --- | --- | --- | --- | --- | --- | --- |
| 14 | A64.0713 | *agrifolia* | 22.5 | 38.6 | 61.1 | 36.8 | 63.2 |
| 22 | A64.0713 | *agrifolia* | 41.8 | 5.3 | 47.1 | 88.7 | 11.3 |
| 15 | A64.0713 | *agrifolia* | 21.3 | 14.9 | 36.2 | 58.8 | 41.2 |
| 81 | A64.1304 | *cerris* | 24.1 | 39 | 63.1 | 38.2 | 61.8 |
| 82 | A64.1304 | *cerris* | 21.8 | 34.1 | 55.9 | 39.0 | 61.0 |
| 251 | A65.0013 | *chrysolepis* | 19.1 | 9.8 | 28.9 | 66.1 | 33.9 |
| 250 | A65.0013 | *chrysolepis* | 24.2 | 10.8 | 35 | 69.1 | 30.9 |
| 30 | A64.1324 | *coccifera* | 24.5 | 26.9 | 51.4 | 47.7 | 52.3 |
| 31 | A64.1324 | *coccifera* | 16.3 | 17 | 33.3 | 48.9 | 51.1 |
| 261 | A68.0355 | *crassifolia* | 52.9 | 21.5 | 74.4 | 71.1 | 28.9 |
| 261 | A68.0355 | *crassifolia* | 52.9 | 21.5 | 74.4 | 71.1 | 28.9 |
| 205 | A64.1274 | *douglasii* | 73.1 | 22.2 | 95.3 | 76.7 | 23.3 |
| 215 | A64.0406 | *douglasii* | 51.7 | 35.6 | 87.3 | 59.2 | 40.8 |
| 228 | A65.0011 | *engelmannii* | 20.7 | 3.2 | 23.9 | 86.6 | 13.4 |
| 230 | A65.0011 | *engelmannii* | 23.2 | 4.7 | 27.9 | 83.2 | 16.8 |
| 102 | A71.0132 | *garryana* | 18.8 | 51.4 | 70.2 | 26.8 | 73.2 |
| 99 | A71.0132 | *garryana* | 15.1 | 50.3 | 65.4 | 23.1 | 76.9 |
| 100 | A71.0132 | *garryana* | 15.9 | 41.7 | 57.6 | 27.6 | 72.4 |
| 233 | A63.0002 | *grisea* | 21.4 | 17.3 | 38.7 | 55.3 | 44.7 |
| 234 | A63.0002 | *grisea* | 19.4 | 10.6 | 30 | 64.7 | 35.3 |
| 33 | A64.1326 | *ilex* | 19.7 | 2.4 | 22.1 | 89.1 | 10.9 |
| 53 | A64.1326 | *ilex* | 23.6 | 7.3 | 30.9 | 76.4 | 23.6 |
| 44 | A64.1315 | *ilex* | 18 | 0.1 | 18.1 | 99.4 | 0.6 |
| 292 | A64.1184 | *macrocarpa* | 20.2 | 5.6 | 25.8 | 78.3 | 21.7 |
| 293 | A64.1184 | *macrocarpa* | 24.7 | 41.1 | 65.8 | 37.5 | 62.5 |
| 256 | A68.0354 | *oleoides* | 17.2 | 14.3 | 31.5 | 54.6 | 45.4 |
| 257 | A68.0354 | *oleoides* | 45.3 | 0.1 | 45.4 | 99.8 | 0.2 |
| 54 | M05.8009 | *phillyreoides* | 13.3 | 1.6 | 14.9 | 89.3 | 10.7 |
| 56 | M05.8007 | *phillyreoides* | 16.2 | 9.1 | 25.3 | 64.0 | 36.0 |
| 57 | M05.8008 | *phillyreoides* | 12.4 | 0.3 | 12.7 | 97.6 | 2.4 |
| 207 | A63.0007 | *pungens* | 12.8 | 36.4 | 49.2 | 26.0 | 74.0 |
| 235 | A63.0007 | *pungens* | 60.8 | 0.1 | 60.9 | 99.8 | 0.2 |
| 306 | A64.1311 | *robur* | 27.6 | 14.5 | 42.1 | 65.6 | 34.4 |
| 307 | A81.0414 | *robur* | 22.7 | 53.8 | 76.5 | 29.7 | 70.3 |
| 26 | A65.0838 | *rugosa* | 12.7 | 5.2 | 17.9 | 70.9 | 29.1 |
| 258 | A68.0360 | *rugosa* | 38.1 | 8.2 | 46.3 | 82.3 | 17.7 |
| 68 | A64.0062 | *sinuata* | 60.8 | 0.5 | 61.3 | 99.2 | 0.8 |
| 69 | A64.0062 | *sinuata* | 29.4 | 22.5 | 51.9 | 56.6 | 43.4 |
| 65 | A64.1325 | *suber* | 20.9 | 1.9 | 22.8 | 91.7 | 8.3 |
| 79 | A64.1325 | *suber* | 19.3 | 11 | 30.3 | 63.7 | 36.3 |
| 80 | A64.1280 | *suber* | 40.5 | 14.9 | 55.4 | 73.1 | 26.9 |
| 32 | A68.0609 | *tomentella* | 23.8 | 18.7 | 42.5 | 56.0 | 44.0 |
| 32 | A68.0609 | *tomentella* | 23.8 | 18.7 | 42.5 | 56.0 | 44.0 |
| 248 | A64.1309 | *trojana* | 34.9 | 39.2 | 74.1 | 47.1 | 52.9 |
| 298 | A64.0008 | *trojana* | 42.6 | 45 | 87.6 | 48.6 | 51.4 |
| 264 | A69.0181 | *variabilis* | 23.7 | 72.6 | 96.3 | 24.6 | 75.4 |
| 265 | A69.0181 | *variabilis* | 54.1 | 57.1 | 111.2 | 48.7 | 51.3 |
| 58 | A64.0012 | *virginiana* | 16.9 | 35.7 | 52.6 | 32.1 | 67.9 |
| 60 | A64.0012 | *virginiana* | 16.1 | 0.2 | 16.3 | 98.8 | 1.2 |

**Table S3**. Indication of whether native range data for each of the 22 oak species was acquired from the Global Biodiversity Information Facility (GBIF), from the "Atlas of United States Trees" by Elbert L. Little, Jr. (and other publications), or both. For native ranges estimated by GBIF, occurrence data was first obtained by restricting occurrence data to native range locations soured from the Kew Plants of the World Online database. Additionally, occurrence data were filtered by year (2018-2023) and only preserved specimens with coordinates were included. The get_range() function from the gbif.range package in R was then used to estimate the native range for each species based on these occurrence data. For native ranges acquired by the "Atlas of United States Trees", the shapefile of the native range for each species was downloaded from <https://github.com/wpetry/USTreeAtlas/tree/main.> When a species native range data were available from both GBIF and the "Atlas of United States Trees", climate data were pulled out from each and averaged.

| species | native range locations from Kew Plants of the World Online database | Number of occurrences from GBIF | Native range estimated from GBIF | Native range downloaded from Atlas of United States Trees |
| --- | --- | --- | --- | --- |
| *Q. agrifolia* | California, Mexico Northwest | 60 | yes | yes |
| *Q. cerris* | Albania,  Austria, Bulgaria, Czechoslovakia, East Aegean Is., France, Greece, Hungary, Italy, Kriti, Lebanon-Syria, Romania, Sicilia, Switzerland, Turkey, Turkey-in-Europe, Yugoslavia | 36 | yes | no |
| *Q. chrysolepis* | Arizona, California,  Mexico Northeast, Mexico Northwest, Nevada, New Mexico, Oregon | 98 | yes | yes |
| *Q. coccifera* | Albania,  Algeria, Baleares, Bulgaria, Cyprus, East Aegean Is., France, Greece, Italy, Kriti, Lebanon-Syria, Libya, Morocco, Palestine, Portugal, Sardegna, Sicilia, Spain, Tunisia, Turkey, Turkey-in-Europe, Yugoslavia | 41 | yes | no |
| *Q. crassifolia* | Guatemala,  Mexico Central, Mexico Gulf, Mexico Northeast, Mexico Southeast, Mexico Southwest | 102 | yes | no |
| *Q. douglasii* | California | 25 | yes | yes |
| *Q. engelmannii* | California, Mexico Northwest | 23 | yes | yes |
| *Q. garryana* | British Columbia, California,  Oregon, Washington | NA | no | yes |
| *Q. grisea* | Arizona, Colorado, Mexico Gulf,  Mexico Northeast, Mexico Northwest, Mexico Southwest, New Mexico, Texas | 100 | yes | yes |
| *Q. ilex* | Albania, Algeria, Baleares, Corse, Czechoslovakia, East Aegean Is., France, Greece, Italy, Kriti, Portugal, Sardegna, Sicilia, Spain, Switzerland, Tunisia, Turkey, Yugoslavia | 30 | yes | no |
| *Q. macrocarpa* | Alabama, Alberta, Arkansas,  Connecticut, Illinois, Indiana, Iowa, Kansas, Kentucky, Louisiana, Maine, Manitoba, Maryland, Massachusetts, Michigan, Minnesota, Missouri, Montana, Nebraska, New Brunswick, New York, North Dakota, Ohio, Oklahoma, Ontario, Pennsylvania, Québec, Saskatchewan, South Dakota, Tennessee, Texas, Vermont, Virginia, West Virginia, Wisconsin, Wyoming | 130 | yes | yes |
| *Q. oleoides* | Belize, Costa Rica, Guatemala,  Honduras, Mexico Central, Mexico Gulf, Mexico Northeast, Mexico Southeast, Mexico Southwest | 15 | yes | no |
| *Q. phillyreoides* | China South-Central, China Southeast, Japan, Nansei-shoto | 72 | yes | no |
| *Q. pungens* | Arizona, Mexico Northeast,  New Mexico, Texas | 10 | yes | yes |
| *Q. robur* | Albania, Altay, Austria, Baltic States, Belarus, Belgium, Bulgaria, Central European Rus, Corse, Czechoslovakia, Denmark, East European Russia, Finland, France, Germany, Great Britain, Greece, Hungary, Iran, Ireland, Italy, Kriti, Krym, Netherlands, North Caucasus, North European Russi, Northwest European R, Norway, Poland, Portugal, Romania, Sardegna, Sicilia, South European Russi, Spain, Sweden, Switzerland, Transcaucasus, Turkey, Turkey-in-Europe, Ukraine, Yugoslavia | 465 | yes | no |
| *Q. rugosa* | Arizona, Guatemala, Honduras,  Mexico Central, Mexico Gulf, Mexico Northeast, Mexico Northwest, Mexico Southeast, Mexico Southwest, New Mexico, Texas | 50 | yes | yes |
| *Q. sinuata* | Alabama, Arkansas, Florida, Georgia,  Louisiana, Mexico Northeast, Mississippi, North Carolina, Oklahoma, South Carolina, Texas | 6 | yes | no |
| *Q. suber* | Algeria, Corse, France, Italy, Morocco, Portugal, Sardegna, Sicilia, Spain, Tunisia | 42 | yes | no |
| *Q. tomentella* | California, Mexican Pacific Is.,  Mexico Northwest | 10 | yes | yes |
| *Q. trojana* | Albania, Greece, Italy, Turkey,  Turkey-in-Europe, Yugoslavia | 3 | yes | no |
| *Q. variabilis* | China North-Central,  China South-Central, China Southeast, Japan, Korea, Taiwan, Tibet, Vietnam | 41 | yes | no |
| *Q. virginiana* | Alabama, Florida, Georgia, Louisiana,  Mississippi, North Carolina, South Carolina, Texas, Virginia | 23 | yes | yes |

**Table S4**. Species’ means of native range climate parameters for the 22 oak species that were included in the PCA. Climate variables are total precipitation (pre; mm), minimum temperature (tmn; °C), maximum temperature (tmx; °C), average temperature (tmp; °C), and aridity index (AI).

| species | pre (mm) | tmp (°C) | tmn (°C) | tmx (°C) | AI |
| --- | --- | --- | --- | --- | --- |
| *Q. agrifolia* | 623.1 | 13.7 | 6.6 | 20.9 | 0.40 |
| *Q. cerris* | 755.8 | 11.1 | 6.3 | 15.9 | 0.71 |
| *Q. chrysolepis* | 753.4 | 11.5 | 3.9 | 19.2 | 0.53 |
| *Q. coccifera* | 743.1 | 12.5 | 7.6 | 17.5 | 0.62 |
| *Q. crassifolia* | 1469.3 | 21.9 | 15.5 | 28.4 | 0.86 |
| *Q. douglasii* | 774.3 | 10.8 | 3.0 | 18.5 | 0.45 |
| *Q. engelmannii* | 339.4 | 15.3 | 8.0 | 22.7 | 0.19 |
| *Q. garryana* | 1069.8 | 4.1 | -0.7 | 8.9 | 1.25 |
| *Q. grisea* | 432.3 | 11.7 | 3.3 | 20.1 | 0.26 |
| *Q. ilex* | 763.6 | 11.8 | 7.1 | 16.7 | 0.66 |
| *Q. macrocarpa* | 796.2 | 8.8 | 2.6 | 15.0 | 0.70 |
| *Q. oleoides* | 1121.0 | 20.8 | 14.0 | 27.6 | 0.65 |
| *Q. phillyreoides* | 1346.7 | 15.3 | 10.8 | 19.8 | 1.08 |
| *Q. pungens* | 343.4 | 14.1 | 5.5 | 22.7 | 0.20 |
| *Q. robur* | 665.9 | 6.7 | 2.5 | 10.9 | 0.85 |
| *Q. rugosa* | 595.5 | 14.2 | 5.9 | 22.5 | 0.35 |
| *Q. sinuata* | 923.6 | 18.0 | 11.3 | 24.7 | 0.57 |
| *Q. suber* | 698.9 | 13.0 | 7.9 | 18.0 | 0.58 |
| *Q. tomentella* | 437.8 | 14.2 | 7.3 | 21.2 | 0.25 |
| *Q. trojana* | 903.9 | 9.6 | 4.7 | 14.6 | 0.75 |
| *Q. variabilis* | 1594.1 | 12.5 | 7.9 | 17.1 | 1.49 |
| *Q. virginiana* | 1180.2 | 16.2 | 10.1 | 22.4 | 0.78 |

**Table S5**. Correlations between climate variables prior to PPCA. Climate variables are total precipitation (pre; mm), minimum temperature (tmn; °C), maximum temperature (tmx; °C), average temperature (tmp; °C), and aridity index (AI).

|  | pre | tmp | tmn | tmx | AI |
| --- | --- | --- | --- | --- | --- |
| pre | 1.00 | 0.26 | 0.45 | 0.07 | 0.86 |
| tmp | 0.26 | 1.00 | 0.94 | 0.96 | -0.22 |
| tmn | 0.45 | 0.94 | 1.00 | 0.80 | 0.05 |
| tmx | 0.07 | 0.96 | 0.80 | 1.00 | -0.41 |
| AI | 0.86 | -0.22 | 0.05 | -0.41 | 1.00 |

**Fig. S1.** Non-significant relationships between stemwood sugar concentrations measured on the oak species in the common garden and (a) PC1 (a gradient from colder to hotter temperatures) and (b) PC2 (a gradient from wetter to drier conditions). Points are species’ means, with color and shape indicating leaf habit (orange circle=deciduous, blue square=brevideciduous, green triangle=evergreen).

(a) (b)


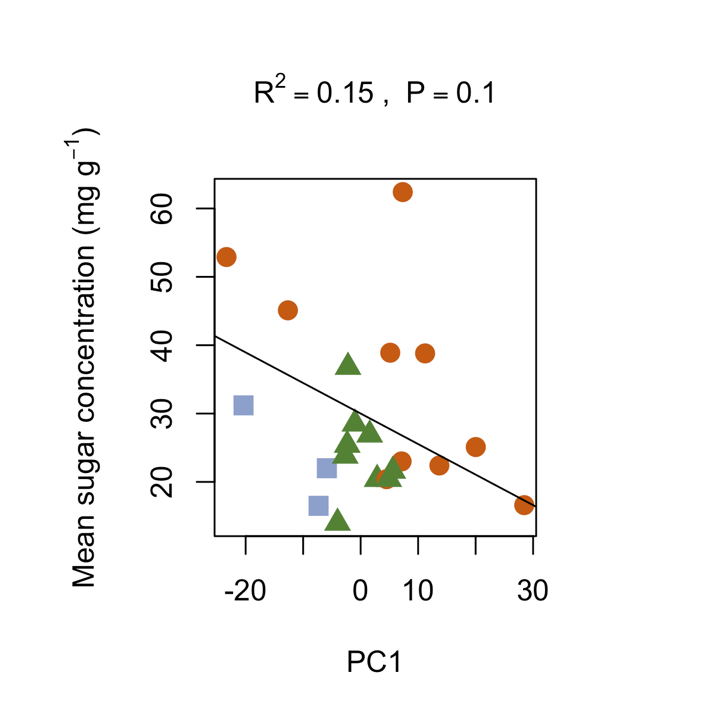

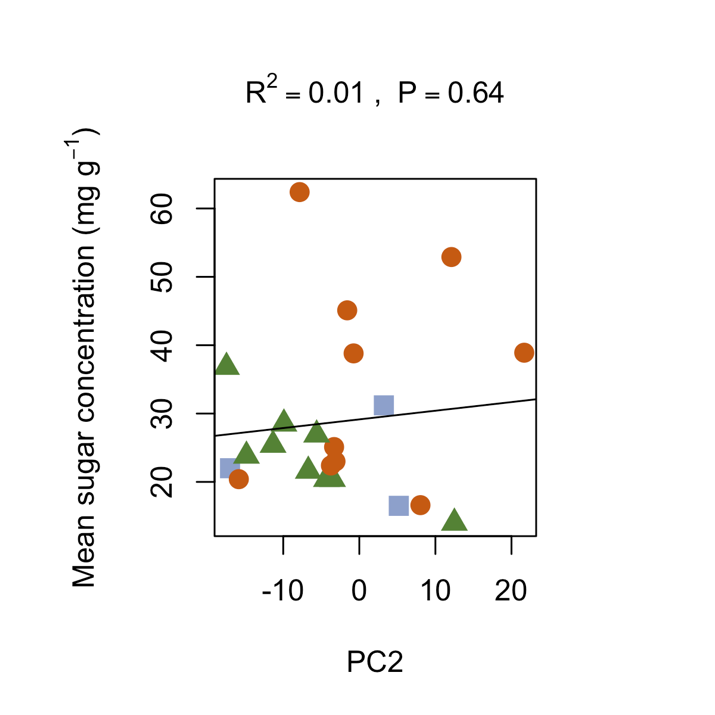

Supplement: Supplementary file 1 — Supplementary file1 (DOCX 170 KB) [file 442_2025_5773_MOESM1_ESM.docx]
